# Anti-Interference Aircraft-Tracking Method in Infrared Imagery †

**DOI:** 10.3390/s19061289

**Published:** 2019-03-14

**Authors:** Sijie Wu, Kai Zhang, Saisai Niu, Jie Yan

**Affiliations:** 1 School of Astronautics, Northwestern Polytechnical University, Xi’an 710072, China; singlechip@163.com (K.Z.); jyan@nwpu.edu.cn (J.Y.); 2 Shanghai Aerospace Control Technology Institute, Shanghai 201109, China; nssycit@163.com

**Keywords:** aircraft tracking, regional distribution, occlusion detection, simulated infrared image

## Abstract

In this paper, we focus on developing an algorithm for infrared-imaging guidance that enables the aircraft to be reliably tracked in the event of interference. The key challenge is to track the aircraft with occlusion caused by decoys and drastic appearance changes resulting from a diversity of attacking angles. To address this challenge, an aircraft-tracking algorithm was proposed, which provides robustness in tracking the aircraft against the decoys. We reveal the inherent structure and infrared signature of the aircraft, which are used as discriminative features to track the aircraft. The anti-interference method was developed based on simulated images but validate the effectiveness on both real infrared image sequences without decoys and simulated infrared imagery. For frequent occlusion caused by the decoys, the mechanism of occlusion detection is exploited according to the variation of the model distance in tracking process. To have a comprehensive evaluation of tracking performance, infrared-image sequences with different attack angles were simulated, and experiments on benchmark trackers were performed to quantitatively evaluate tracking performance. The experiment results demonstrate that our aircraft-tracking method performs favorably against state-of-the-art trackers.

## 1. Introduction

**Figure 1:**
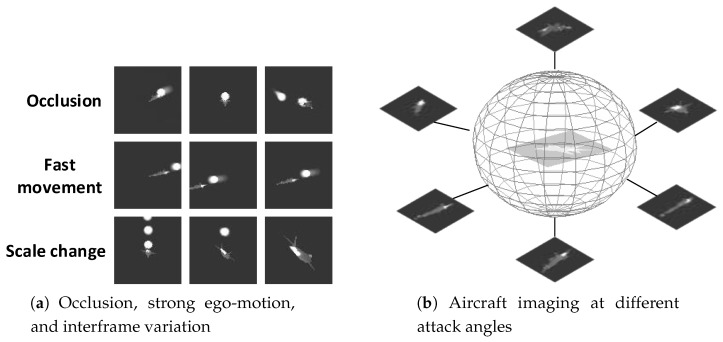
Challenge of aircraft tracking for infrared-imaging guidance.

The detection and tracking of an airborne infrared target in a complex combat environment remain a challenging research field in infrared-imaging guidance [1,2,3]. Infrared technology has been widely used in automatic target recognition because of its all-weather observations. However, in contrast to visual images, infrared images generally have low spatial resolution, poor signal-to-noise ratios (SNR), and lack of textural information [4,5]. In addition, for applications based on an infrared seeker, both the target and the missile are highly maneuverable. The strong ego-motion and scale change dramatically during the approaching process, along with the background cluster, making it more difficult to maintain a robust track [6]. Moreover, due to the extensive use of infrared decoys in the battlefield, to distinguish a target from the infrared decoy through the limited information obtained from the low-resolution infrared images becomes more difficult. The infrared seeker needs to overcome interframe variations resulting from the rapid movement of an aircraft and the occlusion caused by the infrared decoy, as seen from Figure 1. For infrared-imaging guidance, identifying and tracking targets in complex battlefield environments is becoming an increasingly urgent issue.

Although numerous tracking algorithms have been proposed in visual images, only a limited amount of work has been reported on the tracking of targets in infrared images [7,8]. Thermal images are obtained by sensing radiation in the infrared spectrum; due to this property, the signature of IR images is quite different from that of visual images. Visual color patterns and shadows are not available in IR images, and noise characteristics show a significant difference in IR images and visual images [9]. Thus, a tracker that is optimized to handle visual imagery might be suboptimal for IR imagery.

Infrared-imaging missiles process images to derive a target line of sight (LOS) or aim-point [10]. The tracking algorithms for infrared-imaging seekers in the literature range from centroid, edge, and correlation algorithms [11] to those based on more recent computer-vision techniques [12,13,14]. Centroid trackers extract potential targets within an image’s gated area via thresholds and gating techniques and then calculate the first moment of the image intensity [15]. Edge-tracking algorithms track a target by finding the leading edge of the target with respect to its velocity vector [16], and the position of its centroid or edge of interest may be used to determine the aim-point. Correlation trackers maximize the correlation of a reference or “map” within an image frame. They are extremely useful for missile tracking because the bias forces the gate to become fixed on the centroid of the missile nose [15].

For thermal-point tracking, particle- and Kalman filter-based methods have been widely applied [17,18,19]. Teutsch et al. [20] fused three different complementary detection approaches for Kalman tracking, achieving fast and satisfying performance under the assumption that the target motion is linear. For nonlinear and non-Gaussian models, particle filtering was proposed as an alternative to the Kalman filter [21]. Wang et al. [22] combined intensity and edge cues into the particle-filter framework by an adaptive integration scheme. However, the number of particles significantly decreased after several iterations [18]. Zaveri et al. [23] combined particle filtering with interacting multiple-model (IMM) filtering to track an arbitrary target. The use of a mix state also alleviates the degeneracy problem. Lei et al. [24] incorporated the mean-shift algorithm to sample the particles and paid more attention to higher-weight particles. Zhang et al. [25] integrated particle-swarm optimization into the particle-filter framework, transferring particles to a high likelihood region and employing multicue to the likelihood-measured function to improve performance. Tracking algorithms based on particle filters still suffer from a high computational burden.

For target tracking in airborne forward-looking infrared imagery (FLIR), Yilmaz et al. [26,27] extended the mean-shift approach by exploiting the distribution and intensity of the local standard deviation to build a dual-kernel density estimation of the mean shift, providing a general optimization solution. However, a mean-shift-based approach cannot guarantee global optimality, and is susceptible to falling into local maxima in case of clutter or occlusion [28]. When the scale of the target does not significantly vary, tracking can be performed by exploiting morphological operators. Braga-Neto et al. [29] presented a method based on morphological operators for target tracking in FLIR imagery. Morphological connected operators were used in FLIR imagery to extract and track targets of interest and remove undesirable clutter. Bal et al. [30] proposed a tracking algorithm using the intensity-variation function (IVF) and template modeling. IVF was used to capture the target intensity signature. When the IVF was unreliable, the controller triggered the template model to determine the real coordinates of the target. Considering the high ego-motion of the sensor, Loo et al. [31] applied a fringe-adjusted joint transform correlator (JTC) algorithm to compensate the motion, and proposed an enhanced version of synthetic discriminant function (SDF) to update the target model. Dawoud et al. [32] analyzed three main failures modes, ego-motion compensation failure mode, tracking failure mode due to low SNR and background clutter, and the reference-image distortion failure mode, known as the “drifting problem”. Ling et al. [33] applied a performance-evaluation module to decide whether to trigger a position-correction process. Based on previous work, Lamberti et al. [34] exploited a motion-prediction metric to identify the occurrence of false alarms and to control the activation of a template-matching-based phase, thus improving the robustness of target-tracking algorithms. These algorithms work well in a simple combat environment with noninterference, but the performance of the algorithms degrades significantly when processing infrared decoys [35].

With the development of thermal cameras, studies on tracking algorithms have received much attention [36]. Methods based on distribution-field tracking (DFT) [37] rely neither on color nor on sharp edges, which makes it suitable for thermal infrared imagery [38]. The wide basin of attraction around a target’s location also helps in handling uncertainty about the target. Tracking algorithms based on a distribution field also achieve favorable performance in the object-tracking challenge [36,39]. Felsberg [40] used the theoretic connection between distribution fields, averaged shifted histograms, and channel representations to derive an enhanced computational scheme for distribution-field tracking. Furthermore, Berg et al. [38] exploited background information for the online-template update, combining it with a scale-estimation module to improve tracking. Meanwhile, the distribution field can also be integrated into other tracking frameworks to improve performance [41].

For airborne-target tracking based on an infrared-imaging seeker, targets of interest are often noncooperative, and exhibit strong maneuvering action as an evasion tactic [42], and the scale of the target drastically changes during approach. The searching strategy of the DFT tracker is easy to fall into local minima due to the rapid movement of the target, and the fixed scale of the tracking region may not well represent the target. In addition, frequent occlusion caused by infrared decoys leads to the model-drift problem.

Motivated by the above observations, the paper proposes a regional distribution tracking algorithm based on the infrared signatures of the aircraft and the decoy, the main contributions of which can be summarized as follows. First, the searching mechanism of the DFT tracker [37] is improved with region proposals that are formed by clustering the peak values in the equivalent topographic map of the image. Second, the structure information preserved in region proposals is quantified and fused with the original distribution fields to enhance the description capability of the distribution field. Third, an occlusion-detection mechanism is proposed via the variation of the model distance in tracking process to alleviate the model-drift problem. Experiments with different attack angles were conducted to evaluate the performance of the proposed algorithm. The evaluations demonstrate that our tracking algorithm improves the performance of the baseline method and outperforms several other state-of-the-art approaches.

## 2. Distribution Field

**Figure 2:**
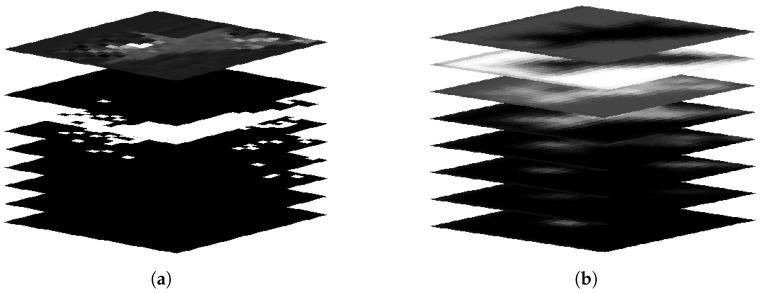
Aircraft distribution. (**a**) number of layers quantized to 8, and original image is superimposed at the top; (**b**) feature maps after smoothing.

First, the distribution field [37] is briefly introduced. The distribution field divides the image into different layers according to the grayscale and assigns corresponding eigenvalues to each layer using the Kronecker delta function. Distribution field $d_{m}\left(i,j,k\right)$ can be denoted as:

(1) $$d_{m}\left(i,j,k\right)=\left\{\begin{array}{c} 1,\;I(i,j)\in [(k-1)*256/m,\;k*256/m),\;\\ 0,\;otherwise, \end{array}\right.$$

where *k* is the layer of the image, and *m* is the stratified number. Visualization of the distribution field is shown in Figure 2a. The spatial domain is processed by a Gaussian filter to deal with uncertainties of the target’s location, as given by:

(2) $$d_{s}\left(k\right)=d\left(k\right)*h_{\sigma s},$$

where $h_{\sigma s}$ is a two-dimensional Gaussian kernel with deviation $\sigma_{s}$ and * stands for convolution operator. Similarly, considering the change of pixel values due to illumination change, fast movement, and occlusion, the feature domain is processed by:

(3) $$d_{ss}\left(i,j\right)=d_{s}\left(i,j\right)*h_{\sigma f},$$

where $h_{\sigma f}$ is a one-dimensional Gaussian kernel with deviation $\sigma_{f}$. After filtering the feature domain and spatial domain, the feature value of each pixel represents probability at a certain layer, which reflects the infrared target’s hierarchical distribution. The final feature maps are shown in Figure 2b.

## 3. Aircraft-Tracking Algorithm

In this section, the tracking algorithm based on region proposals and the distribution field is presented. To effectively describe the characteristics of the target, adequate analysis of the infrared imagery needs to first be done.

### 3.1. Aircraft and Decoy Infrared Signatures

Thermal images are obtained by sensing radiation in the IR spectrum. At temperatures higher than absolute zero, each object emits thermal radiation. The intensity of this radiation depends on the object’s temperature and emissivity [43]. Hot engine parts, exhaust plume, rear fuselage, and aerodynamically heated skin are the important sources of IR emission in an aircraft [44]. In addition, the skin of an aircraft is warm in contrast to the sky background, which makes the aircraft susceptible to detection and tracking by missiles. The decoy, on the other way, is an off-board countermeasure that pulls the track of the missile away from the aircraft by providing a more attractive target [45]. This requires radiating a stronger signal than that of the target in the tracker’s band of interest because many trackers home in on the brightest source [46].

**Figure 3:**
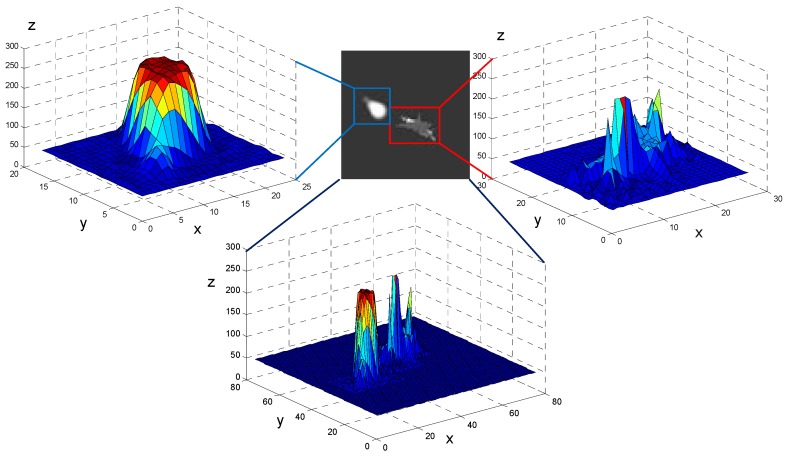
Aircraft and decoy infrared signatures. The figures in the upper left and upper right correspond to the decoy and the aircraft respectively. The infrared signatures show significant diffidence in the aircraft and the decoy. The x, y, and z coordinates represent the row, column, and gray-level values of the image.

Infrared imagery is simulated based on the signature of the aircraft and decoy, as shown in Figure 3. For better visualization of gray-level-value distribution, the gray-level values of the image are converted into their equivalent topographic map. According to Figure 3, the gray-level values of a decoy region’s distribution perform like Gaussian distribution, and the decoy region forms a peak that centers around the combustion and gradually descends to its surroundings. These characteristics are consistent with the description in Ref. [47]. In contrast, the aircraft exhibits multiple peaks according to the radiation characteristics of different regions.

Therefore, considering the above analysis, both texture distribution and shape structure show significant diffidence in the aircraft and the decoy. On this basis, regional distribution is introduced to extract the structure- and texture-distribution characteristics. First, region proposals are generated with the clustering algorithm. Then, the spatial distribution of the region proposals is used to reflect the structural information of the target. After that, the structure feature is fused with the gray-level distribution field [37] to form a region-distribution descriptor. Based on the robust representation of the descriptor, we verified tracking performance in simulated infrared-image sequences.

### 3.2. Region Proposal for Searching

Cluster-based algorithms were successfully applied to the extraction of regions of interest in infrared images [48,49,50]. Among the clustering algorithms, c-means-based methods, including hard c-means and fuzzy c-means, have gained attention and been widely adopted by researchers [51,52,53]. The hard c-means algorithm, also known as k-means algorithm, aims to separate data into c distinct clusters by minimizing the distance from the cluster center [50]. The fuzzy c-means algorithm (FCM) introduces the idea of a membership function and has robust performance for ambiguity when clustering the data [54]. However, the standard FCM algorithm is sensitive to noise and imaging artefacts. Local spatial information and gray-level information were taken into consideration to improve clustering performance [55]. This also increases the computational burden of the algorithm. To improve searching efficiency, the k-means algorithm is considered for its lower computational complexity. Both the k-means and the FCM algorithms suffer from the initialization of clustering centers and the number of clusters. Subsequent analysis shows how to carefully set up the initial centers to accelerate the convergence process and demonstrate comparable performance with other clustering algorithms.

**Figure 4:**
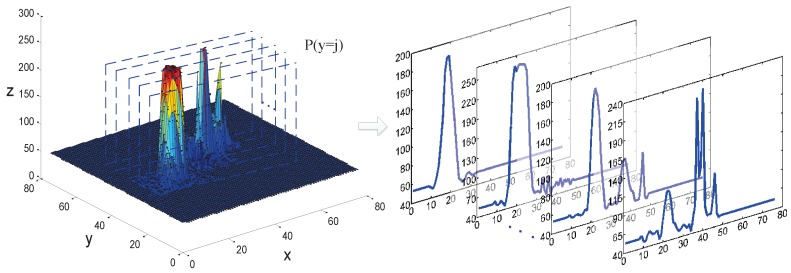
Extraction of image-surface profiles. Assuming the image surface is z(x,y), and p(y=j) is the slice face, as shown in the left figure. The generated surface profiles are shown in the right figure.

According to the preceding analysis in Section 3.1, the target and decoy are high-intensity sources, represented as high-gray-value regions in the imagery. First, the gray-level values of the image are converted into their equivalent topographic map. The number of clusters and clustering centers are assigned by searching the peak values, as shown in Figure 4. This is assuming the image surface is z(x,y), and p(y=j) is the slice face, which is used to obtain the surface profile of the topographic map.

After extracting the surface profiles, the peak values are recorded according to the first-order differential, as defined by:

(4) $$p\left(x\right)=z\left(x+1\right)-z\left(x\right),$$

where $z\left(x\right)$ is the gray value, and the peak value is determined by recording the change of $p\left(x\right)$. Distance measurement between pixels $d_{ij}$ is presented based on the measurement proposed in Ref. [56], as given by:

(5) $$\begin{array}{c} d_{ij}=\frac{||G\left(i,j\right)\;-\;G\left(i_{p},j_{p}\right)|{|}_{1}\;+\;\sigma_{1}||R\left(i,j\right)\;-\;R\left(i_{p},j_{p}\right)|{|}_{2}}{\sqrt{1+{\sigma_{1}}^{2}}}\\ \;\;\;\;\;\;\;\;\;\;\;\;\;\;\;\;\;\;\;+exp(-\frac{G\left(i,j\right)\;-\;G\left(i_{p},j_{p}\right)}{\sigma_{2}}), \end{array}$$

where $i_{p}$ and $j_{p}$ represent each cluster center’s abscissa and ordinate, respectively, *G* and *R* stand for the image’s gray value and measurement in space, and $\sigma_{1}$ is the weight factor to balance the distance measurement. The exponential distance is consistent with the decoy’s distribution, which we discussed before, and $\sigma_{2}$ is the aggregation factor to cluster the pixels of contiguous gray value. Based on the clustering center determined by the peak values, the k-means algorithm [57] was adopted to cluster the pixel to each center.

**Figure 5:**
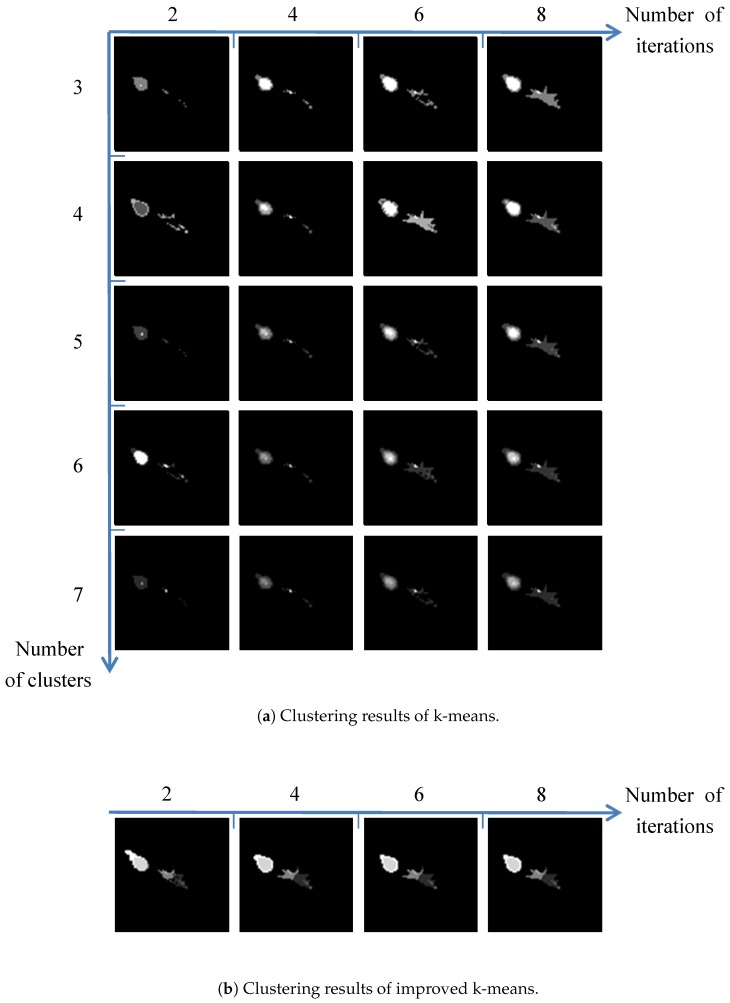
Clustering-result comparison.

The comparison of the cluster results between the improved algorithm and the original k-means algorithm is shown in Figure 5. Each row of Figure 5a stands for the cluster process with the iteration changes, and each column denotes the changes with the number of the clusters. The clustering number of the improved algorithm is 7 in this case, which is determined by searching peak values in the topographic map. As seen from Figure 5b, its convergent process is accelerated compared to the original one.

**Figure 6:**
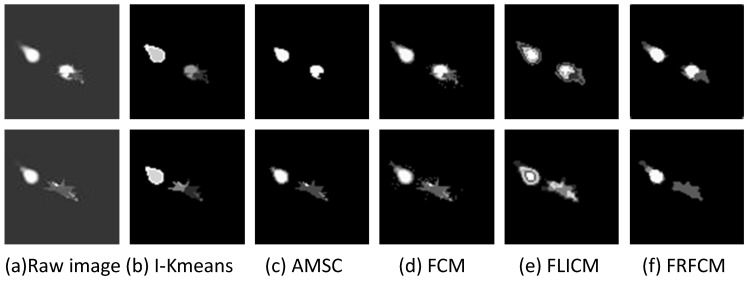
Comparison of different clustering algorithms.

**Figure 7:**
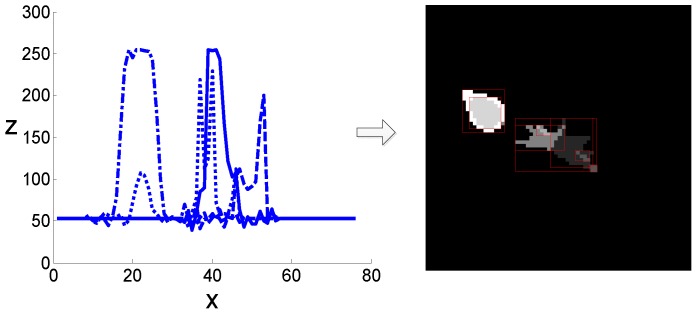
Generation of region proposals. The surface profiles of the image’s topographic map are shown in the left figure. The initial clustering centers are determined by the peak values of the surface profiles. The final cluster result and corresponding region proposals are shown in the right figure.

The comparison between the improved k-means (I-Kmeans) algorithm and other clustering algorithms is shown in Figure 6. The number of clusters is manually set to be consistent with the improved k-means algorithm, except for the mean-shift algorithm, which does not require the setting of an initial number of clusters. As seen from Figure 6, the agglomerative mean-shift clustering (AMSC) algorithm failed to segment the target due to occlusion and the low contrast of the target [58]. The clustering results of the standard FCM algorithm [54] show many isolated clustering pixels, indicating its noise sensitivity. Fuzzy local information c-means (FLICM) [55] much better separates the target from the background, but it is too time-consuming. Fast and robust fuzzy c-means clustering (FRFCM) [59] based on morphological reconstruction and faster membership filtering was proposed to alleviate the computational burden of the FCM algorithm, and did not degrade performance. The improved k-means, the FLICM, and the FRFCM are better to preserve the structural information of the target. Considering the simplicity and efficiency of the algorithm, the improved k-means algorithm was incorporated into the tracking framework. Compared with the results of other algorithms, the cluster results of the improved k-means also indicate the infrared signatures of the aircraft to some extent. The aerodynamically heated skin, rear fuselage, and hot engine parts are clustered to different centers, which is beneficial to subsequent structural analysis of the aircraft. The region proposals were generated on the basis of the cluster result, as shown in Figure 7.

### 3.3. Structural Feature Extraction

This is motivated by the “pictorial structure” proposed by Fischler et al. [60], and then by analyzing the components of the target and measuring their geometric relations. Structural information is proposed based on the distribution of the region proposals.

As shown in Figure 5, the clustering result of the target indicates the structure of the aircraft. The spatial distribution of different patches between the aircraft and the decoy shows significant differences. To describe the spatial information of different patches, we need to encode the spatial coordinates in the appropriate coordinate system. We manually set the center of the patches as the origin of the coordinate system. To more efficiently encode the distance and angle, quantitative analysis of the structural information is performed in polar coordinates. Thus, we can obtain the length and angle coordinates of each patch in the new coordinate system.

**Figure 8:**
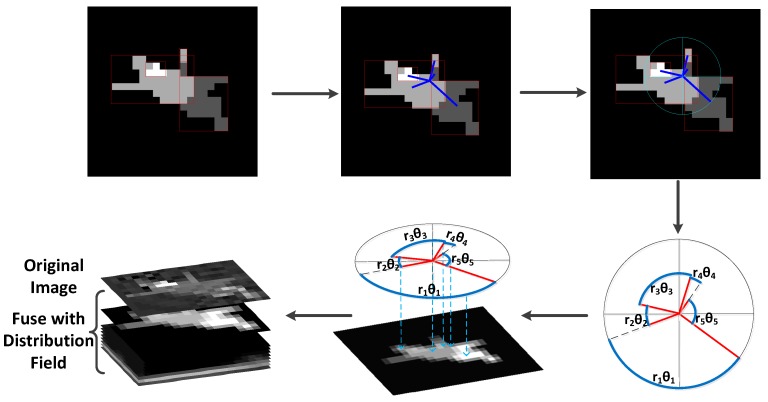
Schematic diagram for generating regional distribution. The structural response values, encoding the relative position between patches, were performed in polar coordinates and mapped to corresponding patches. Then the structural feature map is obtained and integrated into the distribution field to generate regional distribution.

Assume that the regional centroids are $R_{i}\left(x_{i},y_{i}\right),i\in \left(1,...,n\right)$, and $\left\{CR_{1},...,CR_{n}\right\}$ are regional connections, where *C* acts as the regions’ center. The conversion formulas from Cartesian coordinate $\left(x_{i},y_{i}\right)$ to polar coordinate $\left(r_{i},\varphi_{i}\right)$ are defined as follows:

(6) $$x_{c}=(\sum_{i=1}^{n}x_{i})/n\;,\;\;y_{c}=(\sum_{i=1}^{n}y_{i})/n,$$

(7) $$r_{i}=\sqrt{{\left(x_{i}-x_{c}\right)}^{2}+{\left(y_{i}-y_{c}\right)}^{2}},$$

(8) $$\varphi_{i}=\arctan\frac{y_{i}-y_{c}}{x_{i}-x_{c}},$$

where $\varphi_{i}$ and $r_{i}$ stand for the coordinates of angle and radius, respectively. To encode the relative position between patches, we calculated the relative angles according to Equation (9). The *i*-th region’s response value $w_{i}$ is defined in Equation (9). The multiplication of radius and relative angle means the relative arc length, as shown in Figure 8. Thus, we modeled the relative distribution between regions with a scalar value, which encodes both distance and angle information. To introduce spatial uncertainty, a Gaussian filter was used to smooth the structural response values, as given in Equation (10), where $h_{\sigma }$ is the Gaussian kernel with standard deviation σ, and $s_{i}$ is the structural distribution. Then, the structural response values were mapped to the region proposals, as shown in Figure 8:

(9) $$\theta_{i}=\varphi_{i+1}-\varphi_{i}\;,\;w_{i}=r_{i}\theta_{i},$$

(10) $$s_{i}=w_{i}*h_{\sigma }.$$

The geometric structure of the target can be reflected by the region layout. After mapping the response values to region proposals, the structural feature map is obtained and integrated into the distribution field to generate regional distribution, as shown in Figure 8.

**Figure 9:**
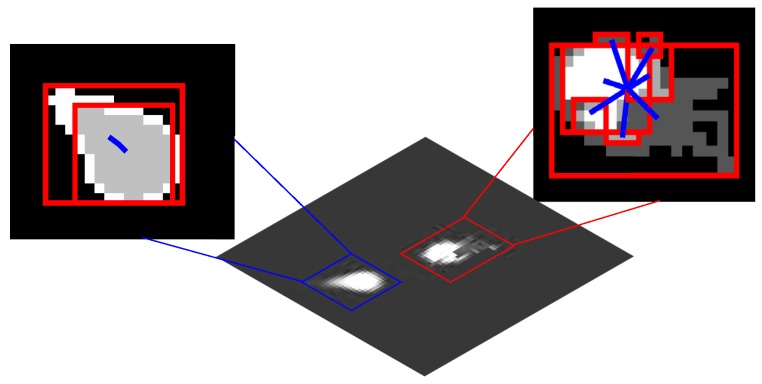
Comparison of structural features.

Regional distribution encodes both gray distribution and structural distribution to enhance the feature descriptor. The structural feature complements the distribution field and helps discriminate the target between the candidate regions. The occlusion case is shown in Figure 9; since the aircraft is partially occluded by the decoy, the distribution field fails to distinguish the target. The structural feature, on the other side, builds up the structural configurations of the target and coordinates with the gray-distribution feature to recognize the target.

### 3.4. Model Matching

After modeling the distribution characteristics of the target, it is necessary to measure the similarity of regional distribution among region proposals. The similarity of the distribution field is defined by

(11) $$\begin{array}{c} L_{1}\left(d_{1},d_{2}\right)=\sum_{i,j,k}\alpha ||d_{1}\left(i,j,k\right)-d_{2}\left(i,j,k\right)|{|}_{1}\\ +(1-\alpha )||s_{1}\left(i,j\right)-s_{2}\left(i,j\right)|{|}_{1}, \end{array}$$

where d(i,j,k) and s(i,j) stand for the distribution field and structural distribution, respectively. Fusion coefficient α is related to stratified number *m* in the distribution field. For the distance in the same order of magnitude, we set α approximate to 1/m.

### 3.5. Occlusion Handling

**Figure 10:**
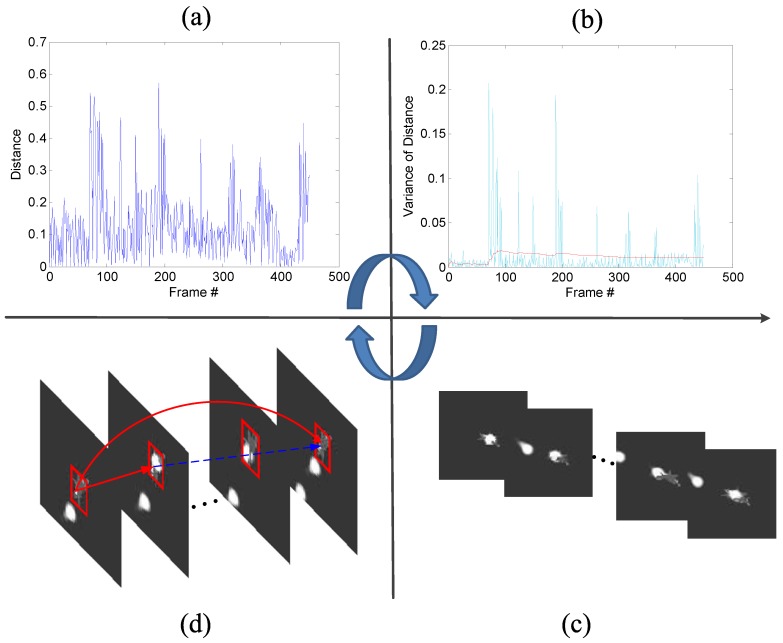
Schematic diagram for occlusion detection. (**a**) distance curve between selected region and target model in the model-matching process; (**b**) red plot represents the statistical variance information of historical frames, and light-blue plot represents the distance bias of the current frame; (**c**) frames that suffer from occlusion corresponding to the peak values in the light-blue plot; (**d**) update process of the model.

Frequent occlusion caused by a decoy increases the difficulty to track the target. When occlusion happens, the target model is contaminated. In this situation, the model is prone to drift without occlusion detection. Based on the model-matching mechanism proposed in Section 3.4, we plotted the distance–variation curve of the target model, as shown in Figure 10a. Since the distance measurement is based on the gray level of the image, the distance has no unit.

Furthermore, to better observe the influence of occlusion, the mean value and variance value of the distance were used to judge the degree of occlusion. In the *n*-th frame, given the previously obtained targets, let $D_{i}$ denote the distance between frame i−1 and frame *i*, i∈(2,...,n). Mean value $\mu_{n-1}$ and variance value $\sigma_{n-1}^{2}$ is updated as follows:

(12) $$\mu_{n-1}=\frac{1}{n-1}\sum_{i=2}^{n}D_{i-1},$$

(13) $$\sigma_{n-1}^{2}=\frac{1}{n-1}\sum_{i=2}^{n}{\left(D_{i-1}-\mu_{i-1}\right)}^{2}.$$

Variance value $\sigma_{n}^{2}$ of the current frame is defined as

(14) $$\sigma_{n}^{2}={\left(D_{n}-\mu_{n-1}\right)}^{2}.$$

The value of $\sigma_{n}^{2}$ can reflect deviation of the current distance from the mean value of the previous frame. The plots of $\sigma_{n-1}^{2}$ and $\sigma_{n}^{2}$ varying with the frame are shown in Figure 10b. If the value of $\sigma_{n}^{2}$ exceeds the value of $\sigma_{n-1}^{2}$, this means that the target undergoes large appearance changes caused by either pose variation or occlusion. In this paper, the related simulation experiments mainly focus on the influence of a decoy, and the aircraft maneuver is reduced. Thus, the target’s pose variation of the adjacent frame does not show significant difference. Therefore, it can be considered that an occlusion is detected, if $\sigma_{n}^{2}$ dramatically changes. Some corresponding frames that suffer from occlusion are listed in Figure 10c. After the tracker enters the occlusion state, the model is updated along on two main lines alone. The tracker saves both the target models of occlusion and nonocclusion. When $\sigma_{n}^{2}$ is down to $\sigma_{n-1}^{2}$, the target model is restored to the preocclusion state, as shown in Figure 10d.

### 3.6. Our Aircraft-Tracking Algorithm

The details of the tracking algorithm are given in Algorithm 1. The initial search regions are determined by region proposals, which are generated based the clustering results. The distribution of each candidate area can be calculated by the regional distribution descriptor, and it updates the model of the aircraft based on the matching mechanism.

**Algorithm 1:** Aircraft-tracking algorithm **Input:** Image sequence. **Output:** Aircraft location with bounding box.  1: Generate region proposals by clustering.  2: **for**
*n* =1 **to**
*m* (*m* is the number of candidate regions) **do**
  3: Calculate gray distribution field.  4: Compute structural distribution.  5: Calculate similarity between target’s model and candidate region.  6: **end for**  7: Detect occlusion via measuring the variation of the model distance.  8: Select a region with the minimal distance as the tracking region.  9: Update the target’s model.

## 4. Experiments and Discussions

### 4.1. Analyzing Regional-Distribution Tracker

#### 4.1.1. Search Space and Feature Representations

**Figure 11:**
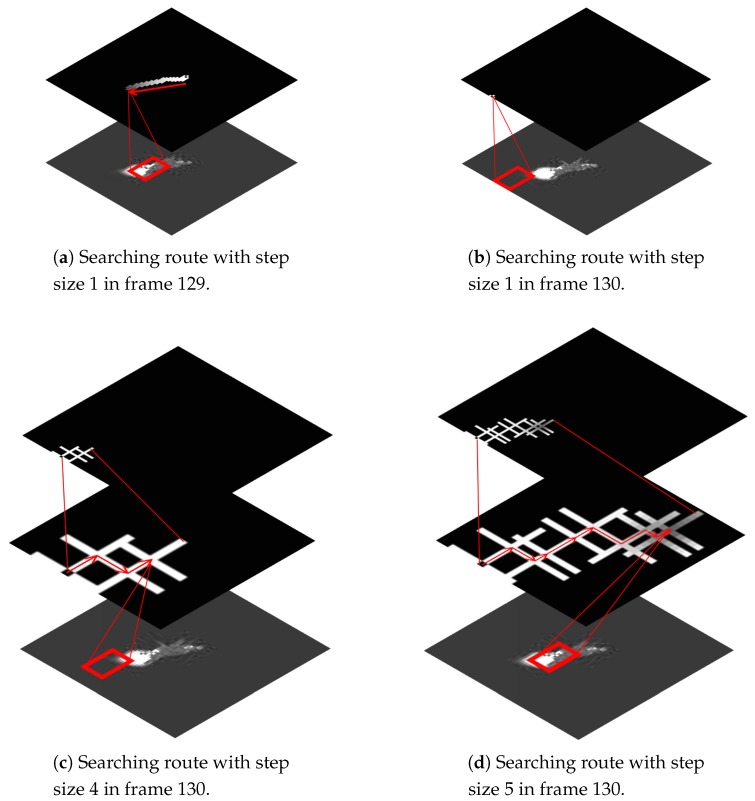
Searching-route comparison.

For the distribution field tracker (DFT) [37], the searching region has a significant impact on tracking performance. To improve searching efficiency, the distribution-field tracker uses motion prediction to compute the start of the search, and adopts a small search step to find the optimal solution. However, when the tracker traps in the local optimum, it is hard to jump from the current position with a small search step, as shown in Figure 11b. We gradually increased the searching step, and compared the tracking results. For more intuitive understanding, the distance maps corresponding to the tracking result are visualized in Figure 11. The gray value of the distance map indicates the distance between the candidate region and the target model, and the searching routes follow the direction of the distance descend, as marked with red arrows in Figure 11. Each cross-shaped region in the distance map represents an iteration of the searching process. For a better view, the search regions were zoomed, and the sketch maps of the distance descend path were superimposed on the original maps after searching. The increase of the searching step helps the tracker converge to the optimal solution, at the cost of more computing time. It should be a trade-off between accuracy and efficiency.

The tracker was evaluated with precision plots and success plots for quantitative analysis [61]. The precision plot shows the percentage of image frames whose tracked location is within the given threshold distance of ground truth. The metric of the success plot evaluates the tracker with bounding box overlap. Given tracked bounding box $B_{t}$ and ground-truth bounding box $B_{gt}$, overlap score *S* and precision *P* are defined as follows:

(15) $$S=\frac{\left|B_{t}\cap B_{gt}\right|}{\left|B_{t}\cup B_{gt}\right|},$$

(16) $$P=\sqrt{{\left(x_{t}-x_{gt}\right)}^{2}+{\left(y_{t}-y_{gt}\right)}^{2}},$$

where $\left(x_{t},y_{t}\right)$ and $\left(x_{gt},y_{gt}\right)$ are the center coordinates of tracked bounding box $B_{t}$ and ground-truth bounding box $B_{gt}$, respectively.

A comparison between the tracking performance of the DFT [37] tracker with default step size 1 and step size 5 is shown in Table 1. Although the increased step size expands the searching scope and improves tracking accuracy, the mechanism of the local search still limits tracking performance. The strong ego-motion makes it difficult to accurately predict the motion, which has a significant impact on the searching region. For comparison, we used region proposals in Section 3.2 instead of the original searching algorithm to test the tracker, i.e., the regional-distribution tracker with original gray-level distribution features (RDT-Gd). Region proposals can be seen as a method of global searching, generated based on the gray-value distribution of the infrared imagery. As illustrated in Table 1, the growing searching region alleviates the target’s fast movement to some extent, so as to improve tracking performance.

The results of the experiments verify the effectiveness of the improved searching algorithm. Next, we performed experiments on both the gray-level distribution feature and regional-distribution feature to validate the performance of the aircraft-tracking algorithm. RDT represents the tracker with a region-distribution feature, which includes gray-level-value distribution and the spatial distribution of subregions. DFT stands for the original distribution field tracker in accordance with the author’s paper [37]. As shown in Table 1, RDT shows the best tracking performance.

#### 4.1.2. Distance Normalization

**Figure 12:**
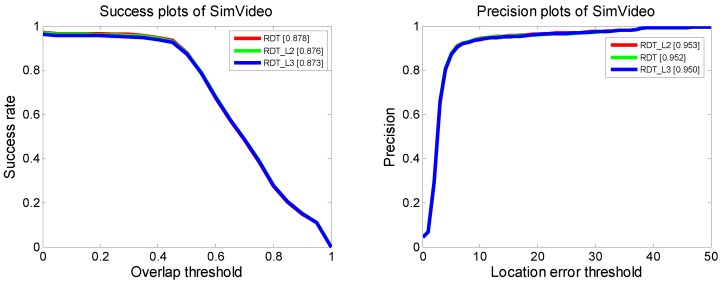
Overall performance of the Regional-Distribution Tracker with different normalization.

**Figure 13:**
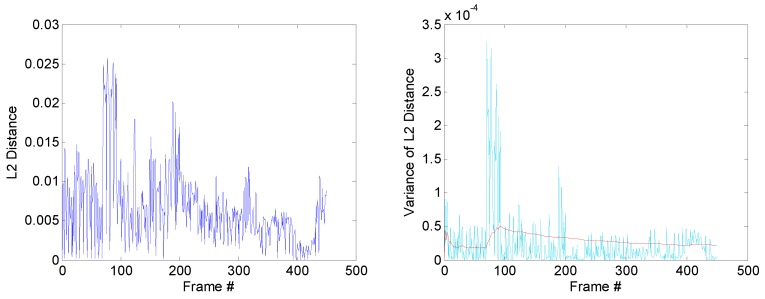

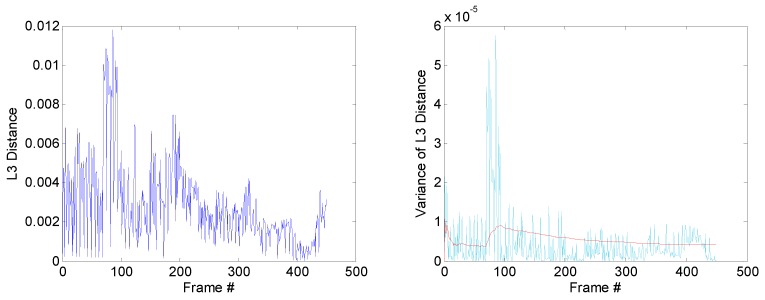
Distance curve with different normalization. The left figures are distance curves between selected region and target model in the model-matching process. The variance information of the distance curve are shown in the right figures. The red plots represent the statistical variance information of historical frames, and light-blue plots represent the distance bias of the current frame.

We followed the distance measurement in the original DFT tracker [37]. For comparison, we also calculated the L2 and L3 norms during model matching. Overall performance is shown in Figure 12. The distance curves between the target region and the selected region are shown in Figure 13. As stated in Section 3.5, we also plotted the distance statistical variance of historical frames and the distance bias of the current frame. The distance margin varied with different normalization, while the main trend of the curves stayed consistent. The overall performance showed a slight difference when using different normalization methods.

### 4.2. Computational-Cost Analysis

**Figure 14:**
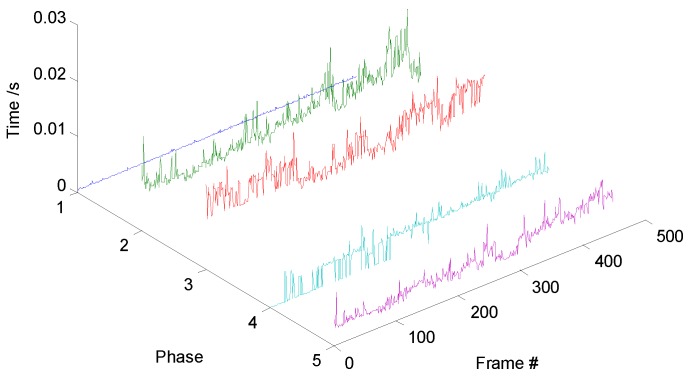
Regional-Distribution Tracker time cost.

**Figure 15:**
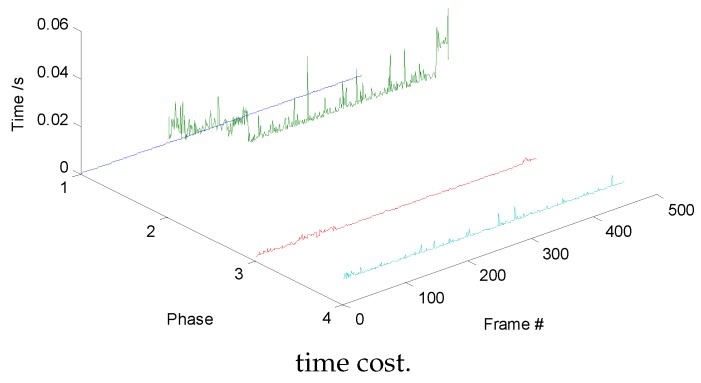
Distribution Field Tracker

The main RDT phase can be divided into five phases, searching peak values, region clustering, gray-level distribution calculation, structural-feature extraction, and model update, corresponding to Phases 1–5; their proceeding time is shown in Figure 14. Similarly, we divided the DFT into four phases. Phases 1–4 in Figure 15 represent motion prediction, distribution-field calculation, search phase, and model update, respectively. Without loss of generality, we chose one of the image sequences to plot the time-cost variation curves during tracking. Gray-value distribution and structural distribution are complementary features during tracking. When the target is far away from the sensor, the structure of the target is hard to be observed, so gray-level distribution plays a major role. As the target approaches, the weight of structural distribution is increased, and the gray-level distribution of the target becomes more complicated; therefore, the clustering phase becomes more time-consuming. The proceeding frames per second (fps) of the RDT and DFT are 26 and 23, respectively, thus improving tracking performance and not degrading the efficiency of the DFT.

### 4.3. Evaluating Tracking Benchmark

**Figure 16:**
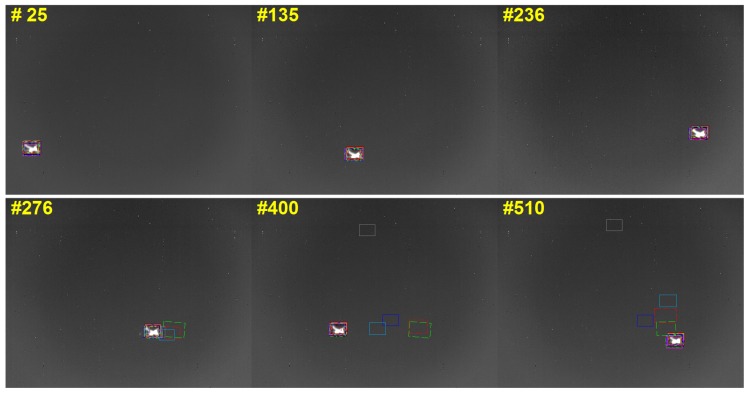
Tracking results of real infrared sequences.

**Figure 17:**
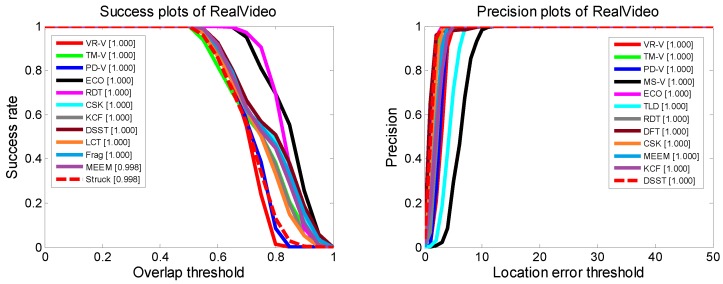
Success plots and precision plots of real infrared sequences.

To evaluate the performance of our aircraft-tracking algorithm, experiments were performed on both real infrared-image sequences and simulated infrared imagery. The tracking benchmark code library [61] was used to test the trackers. We added five state-of-the-art trackers [62,63,64,65,66] to complement the tracking library. For comparison, we first tested the trackers in the real infrared-image sequence without decoys. The image sequence was captured by IRCAM Equus 327 KM (Erlangen, Germany). The working band of the infrared camera is 3–5 μm, and the resolution of the camera is 640 × 512. The camera was stationary during the capturing process. When the target was out of the field of view, the camera position was manually adjusted to let the target reappear in the center of the field of view. Since there was no interference to disturb the tracking process, most of the trackers could reliably track the aircraft, as seen from Figure 16 and Figure 17.

**Figure 18:**
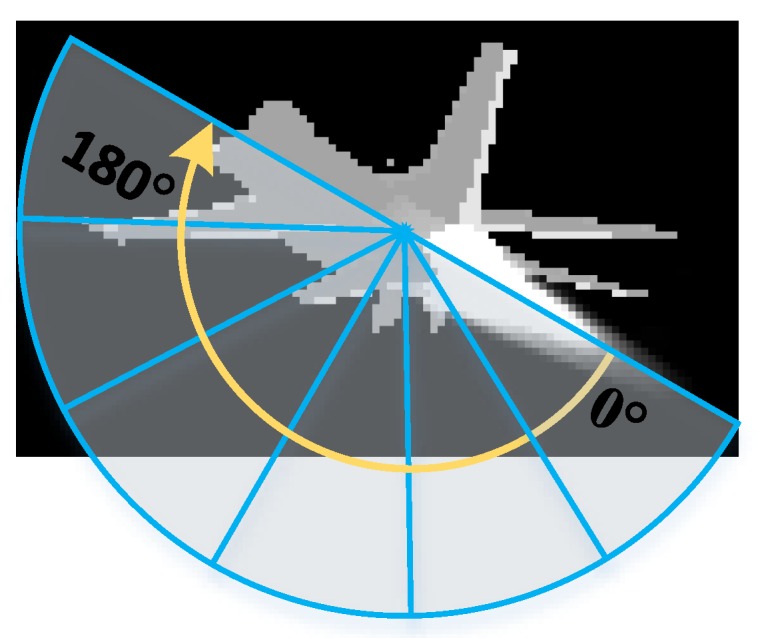
Schematic diagram of attack angle changes. Attack angle varies from 0 to 180 degrees at 30 degree intervals.

**Figure 19:**
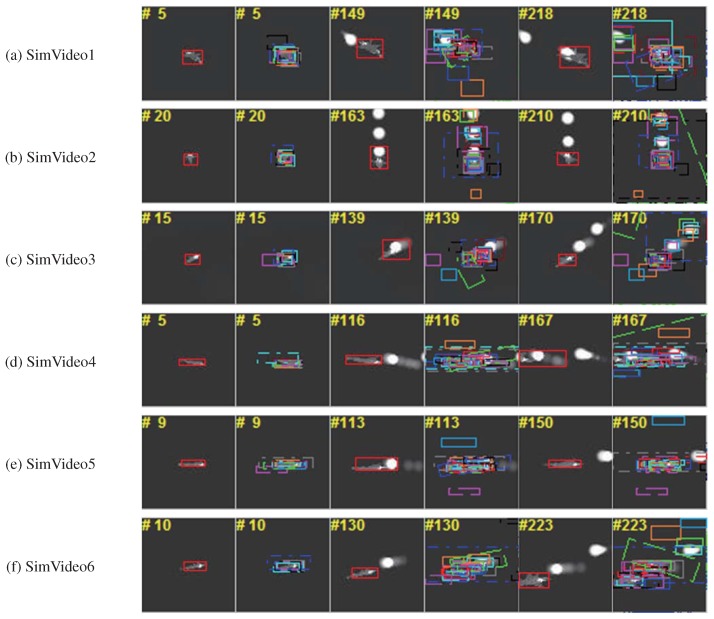
Tracking results of simulated infrared sequences. Our tracker’s results are drawn with a red rectangle for clarity.

Further experiments were conducted on simulated infrared imagery with extensive use of infrared decoys. The simulated environment was developed based on OpenGL and the OpenSceneGraph (OSG) toolkit, which are widely used in the visual-simulation, space, scientific, and virtual-reality industries [67,68,69]. The different parts of the aircraft’s geometric model were rendered based on its corresponding infrared signatures [70,71], as stated in Section 3.1. We simulated the navigation and guidance process of the missile in the generated scene and integrated the tracker to the control system of the missile. The returned position of the target by the tracker was sent to the control system to calculate the attitude angle of the missile, so the missile could fly right toward the target. For an ideal tracker, the target should stay in the center of the field of view. To more comprehensively evaluate the tracking algorithm, infrared-image sequences with different attack angles were simulated. In our experiment, the attack angle varied from 0 to 180 degrees at 30 degree intervals, as shown in Figure 18. The tracking results of the trackers are marked with differently colored bounding boxes, as shown in Figure 19. For clarity, our tracker’s results were drawn with a red rectangle. As seen from Figure 19, most of the trackers could lock onto their target in a simple combat environment without interference, but when the target threw infrared decoys, the trackers significantly drifted and began to track the decoy instead of the aircraft. Compared with the others, our tracker could track the aircraft more effectively.

**Figure 20:**
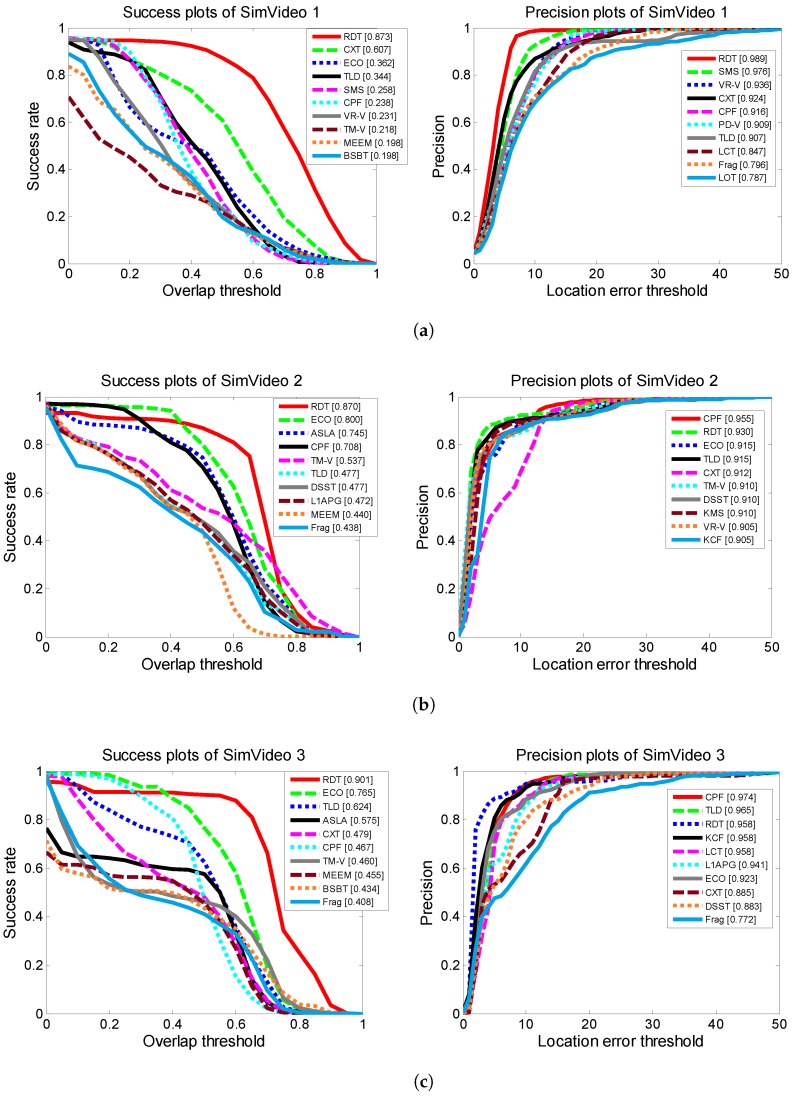

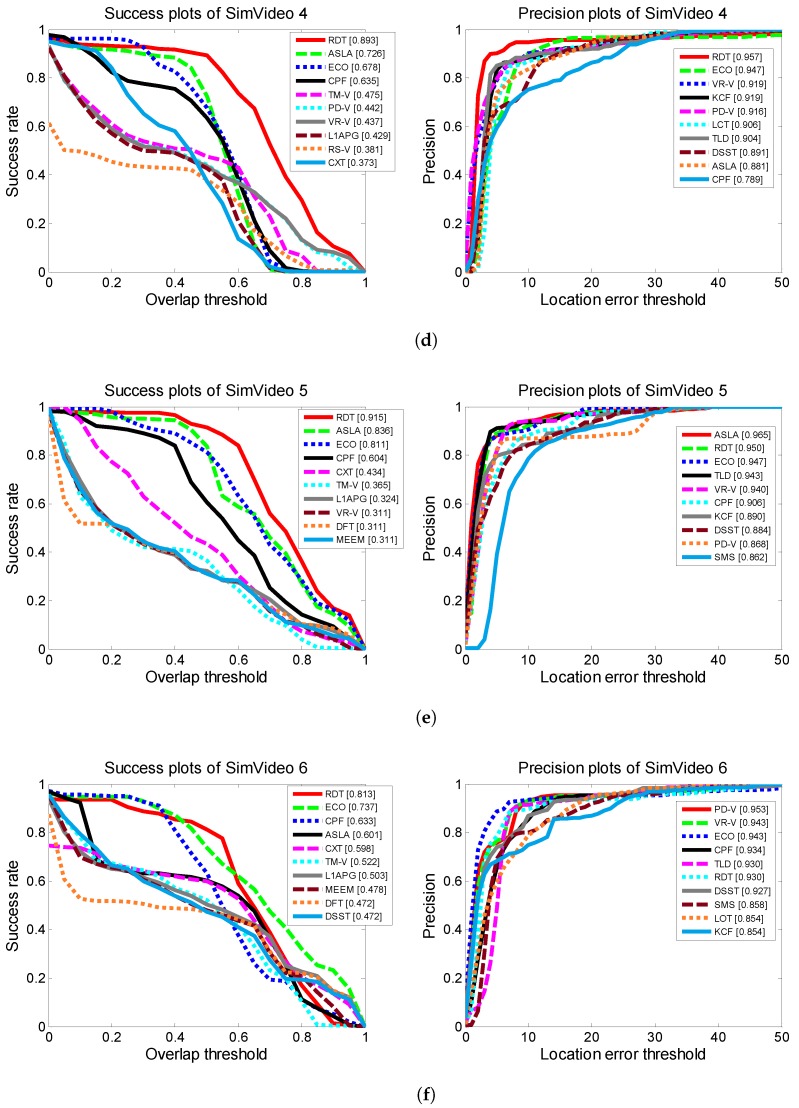
Success plots and precision plots of simulated infrared sequences. The corresponding results of different simulated infrared sequences are shown in subfigures.

The success plots and precision plots of the top 10 trackers in the simulated infrared sequences are shown in Figure 20. Compared with the trackers included in the tracking benchmark code library, the RDT had much better tracking performance than the DFT, and outperformed most of the other trackers.

**Figure 21:**
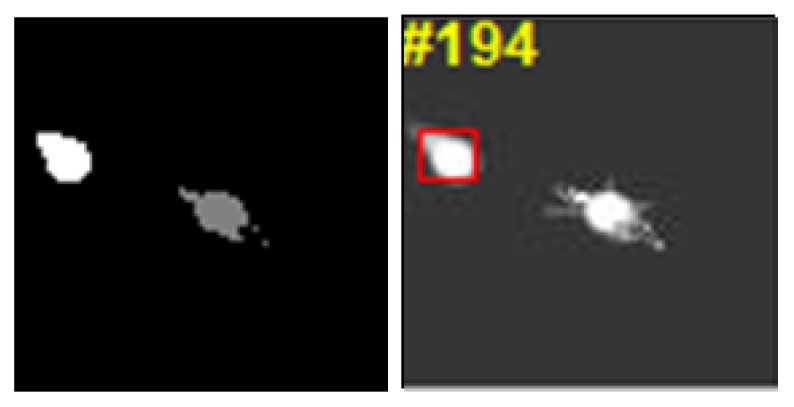
Failure case of tracking results. (**left**) clustering result and (**right**) corresponding tracking result.

Nevertheless, there were still some failure cases with our tracker. Clustering performance had an influence on our tracker. The clustering result and the corresponding tracking result are shown in Figure 21. The decoy occluded the main part of the aircraft, which led to similar clustering results for the aircraft and the decoy. The tracker failed to recognize the aircraft from both the gray-level feature and the structural feature. Therefore, the tracker drifted to the decoy, which we need to improve in the future.

## 5. Conclusions

In this paper, an aircraft-tracking algorithm based on regional distribution in the case of interference was presented. This algorithm improves the searching mechanism of the distribution-field tracker with region proposals, which are generated through exploiting the infrared signatures of the aircraft and the decoy. To enhance the description capability of the feature, we encoded both structural information and gray-level-value distribution to the distribution field. For frequent occlusion caused by decoys, a mechanism of occlusion detection was introduced to ensure the accuracy of the target model. Experiments with different attack angles show that our aircraft-tracking algorithm improves the performance of the baseline method, and performed better than several other state-of-the-art trackers in simulated infrared-image sequences in terms of accuracy and robustness.

For aircraft tracking, both the missile and the target are highly maneuverable. It is important to enlarge the searching and extract regions of interest to concentrate on the possible regions. The coarse-to-fine search strategy helps to lock onto the target even if the target is partially occluded. The generation of region proposals is only applicable to single-channel images and is a challenge for properly processing peak values that occur in multichannels. For air-to-ground applications, the background becomes more complex than in the sky, ground temperature may be higher than the target, and extracting the region of interest could be more challenging.

Future work will include how to properly model the target motion and integrate it to the tracking framework. The prediction of target motion helps guide the generation of region proposals and helps make a decision when the target and interference show similar characteristics, thus improving both efficiency and accuracy.

## Figures and Tables

**Table 1:** Characteristics and tracking results of the distribution-field tracker (DFT), regional-distribution tracker with original gray-level distribution features (RDT-Gd), and RDT.

Trackers	Search Space	Feature Representations	Precision	Success
DFT	Local region with step size 1	Gray-level-value distribution	0.389	0.280
DFT	Local region with step size 5	Gray-level-value distribution	0.446	0.302
RDT-gd	Region proposal	Gray-level-value distribution	0.937	0.770
RDT	Region proposal	Regional distribution	0.952	0.878
